# AI-Based Triage Decision Support: Multisite Economic Evaluation in the United States

**DOI:** 10.2196/95213

**Published:** 2026-06-03

**Authors:** Scott Levin, Ben Steinhart, Rohit Sangal, Lesley Meng, K Davina Frick, Cabrina McGinn, Jeremiah Hinson, Andrew Taylor

**Affiliations:** 1 Danaher Diagnostics, Danaher (United States), 2200 Pennsylvania Avenue NW, Washington, DC, 20037, United States, 1 301-404-7742; 2 Department of Emergency Medicine, School of Medicine, Yale University, New Haven, CT, United States; 3 Department of Operations, School of Management, Yale University, New Haven, CT, United States; 4 Carey Business School, Johns Hopkins, Baltimore, MD, United States; 5 Radiometer, Copenhagen, Denmark; 6 Department of Emergency Medicine, School of Medicine, Johns Hopkins University, Baltimore, MD, United States; 7 Department of Emergency Medicine, School of Medicine, University of Virginia, Charlottesville, VA, United States

**Keywords:** emergency medicine, triage, economic model, artificial intelligence, AI, clinical decision support

## Abstract

**Background:**

Emergency department (ED) visits have risen in the United States, with demand for emergency care exceeding supply. Resultant ED crowding harms patients, causes staff burnout, and places financial strain on hospitals, payers, and patients alike. Digital tools, including those leveraging artificial intelligence (AI), offer promise for driving efficiency and mitigating the harms of crowding. However, economic frameworks for evaluating these tools remain underdeveloped, limiting adoption.

**Objective:**

This study aimed to develop and apply a generalizable economic model to assess the financial impact of AI-driven efficiency (improved patient throughput) in emergency care. The model contrasts hospital management versus public policy cost-modeling approaches.

**Methods:**

We applied an economic model to operational and financial data collected from 3 EDs (170,723 visits across 180-day preintervention and postintervention periods) following the implementation of an AI triage clinical decision support system. Revenue was directly measured, and costs and operating margin were estimated using 2 cost-modeling frameworks: hospital management (accounting for fixed, variable, and modifiable costs) and public policy (cost-per-visit approach). Sensitivity analysis was used to generalize the model to changes in patient throughput spanning −10% to +10% for 2 ED scenarios: capacity-constrained EDs with volume increases and volume-stable EDs. Break-even analyses determined the maximum sustainable AI tool cost per visit under each scenario that would preserve operating margin for each scenario.

**Results:**

Postintervention revenue increased US $15.4 million (US $138.3 million to US $153.7 million) after AI triage clinical decision support implementation and was accompanied by a 9.6% (7797/81,466) increase in ED visit volume. The financial impact on operating margin from this revenue increase differed substantially by cost-modeling framework. Under the hospital management perspective, costs increased US $2.9 million (US $130.3 million to US $133.2 million), attributing most incremental revenue to an operating margin gain (US $12.6 million). Under the public policy perspective, costs rose proportionally with revenue (US $14.5 million; US $130.3 million to US $144.8 million), generating only US $0.9 million in incremental operating margin. This yielded remarkably different break-even thresholds: US $66.02 per visit (hospital management) versus US $4.69 per visit (public policy) for a 5% (16/311) gain in efficiency (patient throughput) in capacity-constrained ED settings.

**Conclusions:**

The financial impact of tools that promote increased ED efficiency varied greatly based on cost-modeling framework. Traditional policy-level approaches substantially underestimate value from a hospital management perspective. The economic model provides a pragmatic and generalized framework for evaluating AI-driven efficiency in the ED, addressing a critical gap that can limit adoption. The economic model is publicly available and adaptable to local contexts.

## Introduction

Annual emergency department (ED) visits have risen to more than 145 million in the United States, with demand for emergency care far outstripping supply [1,2]. Resultant ED overcrowding harms patients, causes staff burnout, and financially burdens hospitals, payers, and patients [2-5]. While patient care impacts have been well studied, ED crowding’s financial consequences have garnered less attention. This is despite ED spending growing faster than other health care sectors [6,7]. Digital tools that leverage artificial intelligence (AI) hold promise for increasing operational efficiency and curbing ED crowding [8-11]. However, a significant gap remains in the economic evaluation of such technologies. Without a practical framework to guide such analyses, health care decision-makers lack the ability to assess cost-effectiveness and financial impact in their local context.

Existing economic evaluations typically apply public policy-level cost perspectives (eg, cost-to-charge ratios) that do not account for the fixed versus variable cost structure central to hospital operations [12-14]. From a hospital management perspective, most ED costs are fixed to maintain 24/7 standby capacity regardless of patient volume [12-14]. Fixed costs include standby labor and facility, equipment, and overhead. The remaining costs are patient throughput–dependent; these include variable costs (eg, supplies and pharmaceuticals) scaling directly with patient volume and modifiable labor costs (eg, contract labor and overtime) that fluctuate with busyness, a product of patient volume and length of stay (LOS) [15-18]. Contract nursing labor has become a particularly significant expense, with disproportionately high use in EDs [17,18]. Under this cost structure, efficiency gains that increase patient throughput and/or reduce care hours delivered can substantially improve operating margins (revenue minus costs) [19]. In contrast, policy-level analyses apply a consistent cost per visit based on hospital charges and cost-to-charge ratios [20,21], which fundamentally differ from hospitals’ true cost structures. This limits applicability to decisions regarding AI-driven ED efficiency tools.

The objective of this study was to develop and apply an economic model to assess the financial impact of AI-driven efficiency (gains in ED patient throughput), contrasting hospital management versus public policy cost-modeling approaches. We applied the economic model to data collected from a multisite AI triage clinical decision support (AI triage CDS) intervention [8]. The economic model is publicly available [22] and easily adaptable to local contexts. The study introduces a novel dual-framework for evaluating AI-based ED interventions and demonstrates that choice of cost-modeling approach can substantially alter estimated financial value, with implications for adoption decisions.

## Methods

### Study Design and Participants

This secondary economic analysis used data from a previously reported preintervention and postintervention study of the AI triage CDS across 3 EDs within a single health system [8]. Implementation occurred in staggered fashion between May 19, 2021, and April 4, 2023. The analysis included 170,723 ED visits during 180-day preintervention and postintervention periods at each site, excluding 2.2% (3925/174,648) of visits in which patients left prior to disposition. Cohort demographics and patient outcomes have been previously reported [8].

### Ethical Considerations

The study received approval from the Yale University Institutional Review Board (2000036009) and is reported in accordance with the Consolidated Health Economic Evaluation Reporting Standards (CHEERS) statement (Checklist 1) [23].

The expected operating margin rate of 5.8% was adjusted from a national report by Wilson and Cutler [19] to account for payer type and admission rate across the study sites.

### AI Triage CDS Intervention

The AI triage CDS [8,9,24-26] applies site-specific machine learning algorithms to ED triage data routinely collected on patient arrival to predict risk of 3 patient outcomes: critical care (defined as in-hospital mortality or intensive care unit admission), emergency surgery (any surgery performed in a dedicated operating room suite within 12 hours of ED disposition), and hospital admission. Predictors include demographics (age and sex), arrival mode, vital signs (heart rate, respiratory rate, oxygen saturation, temperature, and systolic blood pressure), chief complaint, and active medical problems.

Predicted probabilities are translated to recommended acuity levels (1‐5, with lower values indicating higher acuity). Probabilities for critical care and emergency surgery meeting specific thresholds trigger recommendations for highest-risk levels (1 or 2), while probability of hospitalization guides recommendations for lower risk levels (3-5) [8]. The AI triage CDS output appears within seconds in the ED nurse workflow, displaying the recommended triage level with individualized explanations of the recommendation generated using Shapley Additive Explanations values transformed to natural language for each patient. Nurses retain triage decision-making autonomy and may assign acuity levels aligned with or divergent from the AI triage CDS recommendations.

Triage acuity levels dictate early course of care for patients and shape ED operational efficiency [27-29]. High-acuity patients are prioritized for immediate evaluation, while low-acuity patients are commonly diverted to high-efficiency workstreams, such as fast-track or vertical care areas (not requiring an ED bed), where they can be treated quickly. Midacuity patients typically follow traditional ED workflows with longer wait times [27].

### ED Efficiency Outcomes

Postintervention, low-acuity visit assignments (levels 4‐5) increased by a relative 48.2% (from 19,934/83,404, 23.9% to 32,300/91,244, 35.4%), enabling more patients to benefit from streamlined fast-track pathways. Conversely, midacuity (level 3) visits decreased 18.7% (from 40,701/83,404, 48.8% to 36,223/91,244, 39.7%), reducing the proportion of patients in the ambiguous category that typically experiences prolonged wait times [8]. Median ED LOS for all patients decreased 6.1% (19/311.0 min; from 311 to 292 min), with 5% (15.6/311.0 minutes) attributed to the intervention after adjustment for confounders [8]. These efficiency gains created capacity for an additional 9.6% (7795/81,466) of visits [8].

### Economic Model

The economic model measured total ED revenue (before the intervention and after the intervention) and provided 2 approaches for estimating costs and corresponding operating margin. Revenue was defined as the payment received by the hospital for health care services; this excludes fees for physician-specific services. Costs were defined as expenses incurred by the hospital to provide health care services [20]. Operating margin was defined as revenue minus cost, with the corresponding operating margin rate shown in Textbox 1 [30]. Operating margin indicates profitability of core patient care operations [30,31]. All economic model inputs and assumptions may be adjusted to reflect local hospital characteristics and projections. A spreadsheet (Microsoft Excel) for static calculations and a script (Python version 3.8; Python Software Foundation) to enable additional sensitivity analyses is publicly available for download [22]. Table S1 in Multimedia Appendix 1 provides a guide to support custom model inputs based on published data that have been inflation-adjusted to current value (2025) using the Bureau of Labor Statistics Consumer Price Index for Medical Care [32]. A formal economics analysis plan was not developed prior to this secondary evaluation; the economic model structure, assumptions, and analytic approach are described in this section, and the full model is publicly available.

Textbox 1. **Economic model inputs.**

Revenue

- US $1698 per emergency department (ED) visit (average) based on data collected during the preintervention period

Hospital costs: hospital management level

- US $1600 per ED visit (average) based on an expected 5.8% operating margin rate on revenue data collected during the preintervention period
- The expected operating margin rate of 5.8% was adjusted from a national report by Wilson and Cutler [19] to account for payer type and admission rate across the study sites.
  - Fixed costs (baseline), 70%
    - Labor, 60%
    - Facility, 5%
    - Equipment (including digital), 3%
    - Overhead (administration), 5%
  - Variable costs, 20%
    - Supplies, 12%
    - Pharmaceuticals, 8%
  - Modifiable costs, 10%
    - Modifiable labor (eg, contract labor and overtime), 10%

Hospital costs: public policy level

- Costs dependent on ED visit volume. Does not account for care hours provided or associated labor.

Operating margin, US $, %

- US $98 per ED visit (average), represented as the difference between revenue and cost at baseline for an expected margin rate of 5.8%: (revenue − costs)/revenue

### Revenue Data

Hospital revenue data for the cohort were collected from billing tables housed in the electronic health record system. Revenue for ED discharged patients was attributed wholly to the ED, while revenue for hospital admitted patients was calculated as a fraction of the revenue realized from their entire hospital stay [19]. This fraction was calculated as 24 hours divided by total hospital LOS (hours) for hospital stays exceeding 24 hours, and 12 hours divided by total LOS for stays under 24 hours. To mitigate the influence of outliers common in revenue data, revenue per ED visit was capped at the 95th percentile (US $7241). This approach purposefully yields conservative estimates of total revenue and operating margin by excluding potential high-revenue outliers, which follow high-acuity emergency pathways independent of triage methods. For patients with missing revenue estimates (5447/170,723, 3.19%), values were imputed using the mean total revenue within strata defined by admission status (discharge or admit) and primary payer type (Medicare, Medicaid, private insurance, and self-pay).

### Cost and Operating Margin Estimates

Baseline preintervention cost was estimated by presuming a 5.8% operating margin rate on observed revenue. This rate was derived for our study cohort based on payer-specific margin rates reported by Wilson and Cutler [19], weighted by the distribution of revenue across payer and admission groups in our preintervention data (Textbox 1). Thus, the total preintervention cost was calculated as total revenue multiplied by (1–0.058). The resulting preintervention total costs and average cost per ED visit served as the reference for postintervention comparisons. Postintervention operating margin was calculated as revenue minus cost.

Two distinct cost frameworks were applied to postintervention data. The hospital management perspective accounts for time and resources, allocating 70% of total costs as fixed for standby ED care, 20% as variable costs dependent on patient volume, and 10% as modifiable clinician labor that fluctuates with care hours (Textbox 1). This allocation is consistent with prior ED cost analyses [12-14] and reflects that most ED costs—including standby capacity labor, facility, equipment, and overhead—accrue independent of patient throughput, while supplies and pharmaceuticals vary with volume, and a smaller portion of labor can be flexed through mechanisms such as contract labor [18], overtime, or flexible staffing. Though exact ratios may vary by institution, the model allows adjustment of these assumptions. Alternatively, the public policy perspective applies a consistent cost-per-visit approach based on hospital charges and cost-to-charge ratios, holding average cost per ED visit constant regardless of time and resources consumed (ie, care hours).

### Sensitivity Analyses

The economic model was applied to preintervention and postintervention data using measured revenue and both cost frameworks (hospital and public policy) to estimate costs and operating margin changes relative to baseline (before intervention). Sensitivity analyses were performed on these results to both conceptualize and generalize how changes in ED efficiency (ED LOS and patient throughput) translate to financial metrics under 2 common scenarios: (1) *scenario 1, capacity-constrained EDs*, where efficiency gains create new capacity for additional patient volume, applicable where demand for ED services exceeds supply; and (2) *scenario 2, volume-stable EDs*, where efficiency gains do not increase patient volume, applicable where demand for ED services is being met.

For both scenarios, ED LOS changes were modeled from the baseline observed median of 311 minutes to −10% (decreased efficiency, LOS increase to 342 min) to +10% (increased efficiency, LOS decrease to 280 min). Preintervention data (volume, LOS, and revenue) served as baseline comparators. Revenue, costs, and operating margin were estimated across this parameter space.

Break-even analyses were conducted to determine maximum AI tool cost per ED visit to achieve break-even, the point at which tool costs would exactly offset operating margin gains. Break-even estimates per visit were calculated as the difference in margin from the preintervention period to the postintervention period divided by total postintervention visits. This represents the per visit cost an efficiency-enhancing intervention could incur without negating the observed operating margin improvement.

## Results

### Financial Impact of the AI Triage CDS

#### Overview

Postintervention revenue increased US $15.4 million (from US $138.3 million to US $153.7 million), driven by a 9.6% (7795/81,466) increase in ED visit volume from 81,464 to 89,259 visits (Table 1). The additional volume was accommodated without expansion of physical infrastructure or staffing, consistent with efficiency gains previously attributed to the intervention after adjustment for confounders. Median ED LOS decreased 19 minutes (15.6 min after adjusting for confounders) [8], during which the EDs accommodated 7795 additional visits.

**Table 1.** Preintervention and postintervention financial metrics.

Metric	Preintervention	Postintervention	Difference
ED^a^ operations			
Patient volume, n^b^	81,464	89,259	7795
ED LOS^c^ (min), median (IQR)^b^	311 (182-554)	292 (173-496)	19
Hospital management level			
Revenue (US $^b^)	138,309,296	153,747,123	15,437,827
Costs (US $^d^)	130,287,357	133,155,246	2,867,889
Operating margin (US $), %	8,021,939 (5.8)	20,591,878 (13.4)	12,569,938
Public policy level			
Revenue (US $^b^)	138,309,296	153,747,123	15,437,827
Costs (US $^d^)	130,287,357	144,829,790	14,542,433
Operating margin (US $), %^d^	8,021,939 (5.8)	8,917,333 (5.8)	895,394

a ED: emergency department.

b ED operations metrics and revenue measures were observed from the artificial intelligence triage clinical decision support intervention study.

c LOS: length of stay.

d Cost and operating margin were estimated by the economic model.

Average ED revenue per visit was US $1698 (SD US $1834) before intervention and US $1722 (SD US $1843) after intervention (Textbox 1), comparable to published reports (Table S1 in Multimedia Appendix 1). The baseline 5.8% operating margin rate, derived from payer-specific margins weighted by our cohort’s revenue distribution, yielded preintervention costs of US $130.3 million (Table 1) and an average cost per visit of US $1600 (Textbox 1). From this baseline, the financial impact of efficiency gains during the postintervention period differed widely based on cost-modeling framework.

#### Hospital Management Perspective

Costs increased modestly by US $2.9 million (US $130.3 million to US $133.2 million), reflecting the high fixed costs (70%) and modifiable costs (10%) that were unchanged after intervention. This cost structure attributed most incremental revenue (US $12.6 million of the US $15.4 million) to operating margin gain. The operating margin rate improved from 5.8% ($8,021,939/$138,309,396) to 13.4% ($20,591,878/$153,747,123; Table 1).

#### Public Policy Perspective

Costs increased proportionally with revenue by US $14.5 million (US $130.3 million to US $144.8 million) as the constant cost-per-visit approach holds average costs fixed regardless of efficiency. This yielded a minimal US $0.9 million gain in operating margin (from US $8.0 million to US $8.9 million), with the operating margin rate unchanged ($8,021,939/$138,309,396, 5.8%).

### Economic Model Sensitivity Analyses

#### Overview

**Figure 1.**
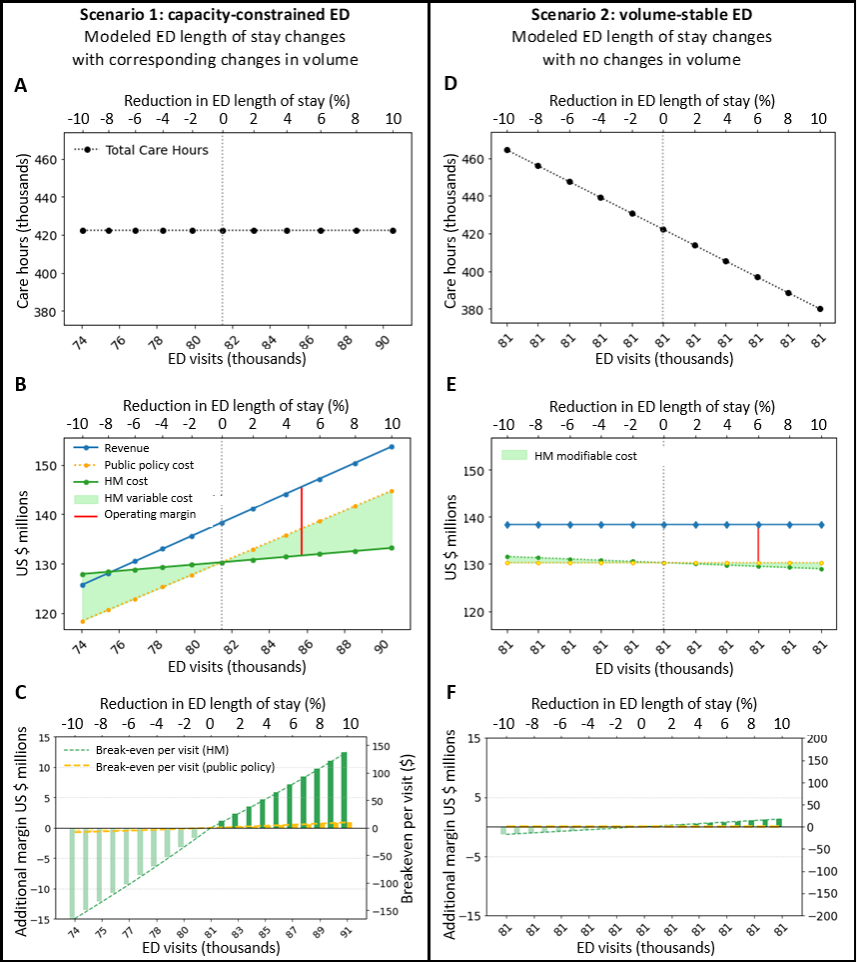
Economic model sensitivity analyses. ED: emergency department; HM: hospital management.

Figure 1 depicts sensitivity analysis results for capacity-constrained and volume-stable ED scenarios.

In Figure 1, changes in ED LOS computed from −10% reduction in efficiency (increase in LOS to 342 min) through a 10% increase in efficiency (decrease in LOS to 280 min). Scenario 1 simulates changes in ED LOS with a corresponding (equal and inverse) change in ED visit volume. Scenario 2 simulates changes in ED LOS with no resulting change in ED visit volume. Hospital management is abbreviated as Hosp. Mgmt.

#### Scenario 1: Capacity-Constrained EDs

In capacity-constrained settings where demand exceeds supply, changes in ED efficiency (LOS) inversely correspond to patient volume changes, holding total care hours (product of patient volume and LOS) constant (Figure 1A). Figure 1B displays the impact on operating margin under the hospital management versus public policy level cost structures. Under the hospital management perspective, operating margin changes substantially with efficiency. Alternatively, the public policy perspective demonstrates costs moving proportionally with volume, minimally impacting operating margin. Contrasting each perspective, an AI tool that increases efficiency by 5% breaks even at US $66.02 per ED visit from a hospital perspective but may only cost US $4.69 to break-even adopting the policy-level perspective on cost (Figure 1C).

#### Scenario 2: Volume-Stable EDs

In this setting, efficiency is not associated with patient volume but does affect total care hours provided (Figure 1D). There is no impact on revenue (Figure 1E) at baseline. However, changes in total care hours due to LOS often require adjustments in modifiable costs for a hospital. Increased efficiency (decreases in ED LOS) presents an opportunity to reduce modifiable labor costs (eg, contract labor and overtime), resulting in an increased operating margin. Under this scenario, an AI tool that increases efficiency by 5% breaks even at US $8.00 per ED visit when total modifiable costs may be reduced by a commensurate 5% (US $13.0 million to US $12.3 million). Applying the public policy cost-estimation approach, revenue and cost do not change, resulting in a decrease in total operating margin with the introduction of any new cost (ie, cost of the AI tool).

## Discussion

### Principal Results

The study demonstrates a financial impact assessment of an AI intervention designed to drive ED efficiency. The assessment illustrates how financial impact varied substantially based on cost-modeling framework. From a hospital management perspective, accounting for fixed and variable cost structures, the AI triage CDS implementation was associated with significant gains in operating margin, amounting to US $12.6 million (158% increase) in our multisite implementation. Conversely, public policy-level cost approaches substantially underestimated economic value, showing only US $0.9 million (11% increase) in margin gain despite identical revenue increases. The large difference in break-even thresholds between perspectives (US $66.02 vs US $4.69 per visit) has direct implications for implementation decisions and highlights the critical need for appropriate economic frameworks when evaluating ED efficiency interventions for a hospital or the public.

The increased financial value under the hospital management perspective reflects ED cost structure: approximately 70% of costs remained fixed (standby capacity), while only 20% varied with volume (supplies and pharmaceuticals) and 10% represented modifiable labor. This meant US $12.6 million of the US $15.4 million revenue increase translated directly to operating margin improvement. The cost structure assumptions (70% fixed, 20% variable, and 10% modifiable) align with prior ED cost analyses [12-14] and reflect the fundamental economics of emergency care delivery. Modifiable labor costs have become increasingly relevant given that contract nursing labor now represents 40% (2022) [18] of hospital-wide nursing expenses, with even higher use in EDs [17]. Our sensitivity analyses demonstrate that even modest reductions in modifiable costs through improved ED efficiency can generate substantial financial value in volume-stable ED settings.

### Comparison With Prior Work

While previous economic evaluations of ED interventions have documented efficiency gains [6,7,12,19,21], they typically do not explicitly distinguish between hospital management and policy-level cost perspectives. Our economic model highlights how cost-modeling approach (perspective) fundamentally shapes economic value assessment. Recent studies have highlighted the promise of AI tools for improving ED triage accuracy and patient flow [8,9,24], but comprehensive economic analyses comparing cost perspectives have been limited.

### Implications for Practice

Hospital decision-makers should evaluate AI efficiency tools using cost frameworks that reflect operational economics, not policy-level per-visit approaches. In capacity-constrained EDs, even modest efficiency gains can yield substantial operating margin improvements when additional patient volume can be accommodated (Figure 1). In volume-stable ED settings, efficiency gains provide opportunities to reduce modifiable labor costs (eg, contract labor and overtime) where applicable. Although the patient perspective was not explicitly measured in these analyses, the operational efficiency gains (reduced LOS) underlying the modeled financial value are patient-relevant outcomes previously associated with reduced morbidity and improved satisfaction [3,8,9,27]. Efficiency gains that improve operating margin can also reduce patient wait times and crowding-related harms. Policymakers should recognize that traditional cost-per-visit approaches substantially underestimate value to hospitals and may inadvertently discourage adoption of beneficial technologies; reimbursement and incentive policies should account for hospitals’ actual cost structures and local market dynamics. Furthermore, developers of AI tools addressing ED crowding should adopt setting-specific value propositions that reflect differential impact based on ED capacity constraints and revenue-generating opportunity from new patient volume.

### Economic Model Generalizability and Adaptability

The economic model developed in this study is designed for broad applicability beyond the specific AI triage CDS implementation studied. We provide the model publicly [22] with guidance for adapting inputs to local contexts (Table S1 and user guide in Multimedia Appendix 1). Our baseline financial metrics align with other large-scale reports of ED financials (Table S1 in Multimedia Appendix 1), with observed differences attributable to cohort selection, payer mix, admission rates (acuity), and accounting methods for ED ancillary and physician-based services. While our cost allocation assumptions (70% fixed, 20% variable, and 10% modifiable labor) are a synthesis of published analyses, institutions can adjust these parameters to reflect local cost structures. Similarly, baseline operating margins, revenue per visit, and efficiency projections can be customized using institutional data or published benchmarks adjusted for inflation.

The 2-scenario approach (capacity-constrained vs volume-stable) allows decision-makers to model their specific operational context. Most US EDs operate in capacity-constrained environments where demand exceeds supply [1,2], making scenario 1 widely applicable. However, EDs in lower-demand settings or those with excess capacity may better align with the volume-stable scenario 2, where value derives primarily from modifiable cost reductions rather than volume increases. The model accommodates both scenarios and the full spectrum between them.

Overall, to support practical application of the economic model, Table S1 in Multimedia Appendix 1 provides published ED financial benchmarks with a user guide for selecting inputs appropriate to local cohort, payer mix, and ED type, and Table S2 in Multimedia Appendix 1 reports the underlying numerical values across the modeled efficiency range (−10% to +10% LOS change), allowing users to read off expected operating-margin and break-even outcomes at specific efficiency assumptions without rerunning the model. Furthermore, EDs assessing their position on the capacity-constrained–to–volume-stable spectrum may consider a combination of indicators—such as inpatient boarding burden, wait times, ambulance diversion, and rates of patients leaving prior to disposition—interpreted alongside local administrative knowledge, recognizing that no single metric reliably distinguishes the 2 scenarios. The −10% to +10% efficiency range modeled in our sensitivity analyses is intended to encompass the magnitude of LOS and throughput gains that may be associated with AI-based ED interventions in isolation.

### Limitations

This analysis relies on data from a single health system, though revenue estimates align with published national data. The economic estimates also depend on the attribution of volume and LOS changes to the AI triage CDS established in the parent intervention study [8]; to the extent that residual unmeasured confounding contributes to those observed changes, the financial estimates here would be commensurately affected. The analysis focused on ED-specific costs and does not capture downstream hospital financial impacts. A formal patient perspective was not modeled; future economic work should incorporate equity considerations and out-of-pocket cost effects to complement the hospital management and public policy perspectives presented here. The break-even analyses assume costs and benefits remain stable over time and do not account for potential learning curves, technology updates, or changing market conditions. How efficiency tools vary in value across different hospital ownership structures (for-profit, not-for-profit, and public), market competitiveness levels, and regulatory environments were not studied. Specifically, the model parameters reflect the US health care payment system; application to publicly funded or mixed systems (eg, Europe) would require substitution of locally appropriate values. Finally, while we demonstrate substantial financial value under realistic assumptions, individual hospital experience will vary based on local factors, including payer mix, labor markets, competitive dynamics, and operational practices. Future research should validate this framework across diverse health systems and settings with varying levels of capacity constraint, cost structures, ownership types, and market conditions.

### Conclusions

This study demonstrates a pragmatic economic model for assessing ED efficiency interventions, applied to a real-world AI triage CDS implementation. The analysis illustrates how financial impacts differ substantially based on the cost-modeling framework. Traditional policy-level approaches substantially underestimate economic value from a hospital management perspective by not accounting for fixed and variable hospital cost structures. The model, publicly available for local adaptation, provides health care decision-makers with a practical tool that accounts for actual hospital cost behavior. As health care systems increasingly consider AI tools to improve efficiency and address ED overcrowding, economic evaluation frameworks that reflect operational realities are essential for informed adoption decisions.

## Supplementary material

10.2196/95213 Multimedia Appendix 1 Economic model input references and economic model sensitivity analyses.

10.2196/95213 Checklist 1 CHEERS checklist.

## Abbreviations

AI: artificial intelligence
CDS: clinical decision support
CHEERS: Consolidated Health Economic Evaluation Reporting Standards
ED: emergency department
LOS: length of stay
