# Supplementary material for: AI-Based Triage Decision Support: Multisite Economic Evaluation in the United States
Source: J Med Internet Res. 2026 Jun 3;28:e95213. doi: 10.2196/95213 (PMC13233007; doi:10.2196/95213)
Supplement: Multimedia Appendix 1 [file jmir-v28-e95213-s001.docx]

## Supplemental File

### Supplemental Table 1. Economic Model Input References

| **Financial Metric** | **Reference** | **Avg $ per ED Visit**  **(Report Year)** | **Avg $ per ED Visit**  **Current (2025) ^a^** | **Cohort Description** | **Hospital Services** | **Physician Services** | **Inpatient Ancillary Services ^b^** |
| --- | --- | --- | --- | --- | --- | --- | --- |
| **Revenue** | AHRQ MEPS[1,2] | $1,233 (2022) | $1,318 | ED All-Comers | Included | Excluded | Excluded |
|  | Scott[3] | $943 (2016) | $1,188 | ED All-Comers | Included | Excluded | Excluded |
|  | Wilson[4] | $656 (2009) | $1,014 | ED All-Comers | Included | Included | Included (34%) |
|  | UH[5] | $2,032 (2018) | $2,416 | UH Claims, PC Conditions | Included | Included | Included |
| **Financial Metric** | **Reference** | **Avg $ per ED Visit**  **(Report Year)** | **Avg $ per ED Visit**  **Current (2025)** | **Cohort Description** | **Hospital Services** | **Physician Services** | **Inpatient Ancillary Services ^b^** |
| **Cost** | AHRQ[6] | $750 (2021) | $821 | ED Discharges Only | Included | Excluded | Excluded |
|  | Rubin[7] | $530 (2017) | $643 | ED All-Comers | Included | Excluded | Excluded |
|  | Wilson[4] | $605 (2009) | $935 | ED All-Comers | Included | Included | Included (34%) |
| **Financial Metric** | **Reference** | **Operating Margin Rate**  **(Report Year)** | | **Facility** | **Hospital Services** | **Physician Services** | **Inpatient Ancillary Services ^b^** |
| **Operating Margin** | Kaiser[8] | 5.2% (2023) | | Hospital (Including ED) | Included | Excluded | Included |
|  | MEDPAC[9] | 8.7% (2021) | | Hospital (Including ED) | Included | Excluded | Included |
|  | **Wilson**[4] | **7.8% (2009)** | | **ED** | **Included** | **Included** | **Included (34%)** |

Agency for Healthcare Research and Quality (AHRQ), Medical Expenditures Panel Survey (MEPS), Medical Payment Advisory Commission (MEDPAC), Emergency Department (ED), Primary Care (PC), United Health (UH)

1. Estimates are have been inflation-adjusted to current value (2025) using the Bureau of Labor Statistics (BLS) Consumer Price Index (PCI) for Medical Care.[10]
2. Denotes accounting for ancillary services for inpatients (ED patients admitted to the hospital)

**User Guide**

Supplemental Table 1 provides a non-comprehensive review of published ED financial metrics to guide input assumptions for economic model users. Data from large US population-based reports that measured hospital facility financials were included. Financial reports demonstrate some heterogeneity due to the selected cohort and methods of accounting for physician services and ancillary services (laboratory and radiology); specifically ancillary services for patients admitted. The methodological details may be important to consider when interpreting the values in Supplemental Table 1. Generally revenues and operating margins will be increased for EDs that are part of tertiary care centers, with higher rates of hospital admission, and/or patients with private insurance as compared to the national averages reported.[4]

Supplemental Table 2. Economic Model Sensitivity Analysis

| **Scenario 1: Capacity Constrained ED, Modeled ED Length-of-Stay Changes with Corresponding Changes in Volume** | | | | | | | | |
| --- | --- | --- | --- | --- | --- | --- | --- | --- |
| **Panel A** | | | **Panel B** | | | | **Panel C** | |
| % Reduction in Length of Stay | ED Visits | Care Hours | Revenue (Millions) | Public Policy Cost (Millions) | Hosp. Mgmt. Cost (Millions) | Hosp. Operating Margin (Millions) | Breakeven per Visit Hosp. Mgmt. | Breakeven per Visit Public Policy |
| -10% | 73,318 | 418,035 | $124.49 | $117.27 | $127.57 | -$3.08 | -$151 | -$11 |
| -5% | 77,391 | 421,201 | $131.41 | $123.79 | $129.00 | $2.41 | -$72 | -$5 |
| 0% | 81,464 | 422,255 | $138.33 | $130.30 | $130.30 | $8.03 | $0 | $0 |
| 5% | 85,538 | 421,203 | $145.24 | $136.82 | $131.57 | $13.67 | $66 | $5 |
| 10% | 89,611 | 418,035 | $152.16 | $143.33 | $132.78 | $19.38 | $127 | $9 |
| **Scenario 2: Volume-Stable ED, Modeled ED Length-of-Stay Changes with No Changes in Volume** | | | | | | | | |
| **Panel D** | | | **Panel E** | | | | **Panel F** | |
| % Reduction in Length of Stay | ED Visits | Care Hours | Revenue (Millions) | Public Policy Cost (Millions) | Hosp. Mgmt. Cost (Millions) | Hosp. Operating Margin (Millions) | Breakeven per Visit Hosp. Mgmt. | Breakeven per Visit Public Policy |
| -10% | 81,464 | 464,481 | $138.33 | $130.30 | $131.61 | $6.72 | -$16 | $0 |
| -5% | 81,464 | 443,368 | $138.33 | $130.30 | $130.95 | $7.38 | -$8 | $0 |
| 0% | 81,464 | 422,255 | $138.33 | $130.30 | $130.30 | $8.03 | $0 | $0 |
| 5% | 81,464 | 401,142 | $138.33 | $130.30 | $129.65 | $8.68 | $8 | $0 |
| 10% | 81,464 | 380,030 | $138.33 | $130.30 | $129.00 | $9.33 | $16 | $0 |

Sample of numeric values underlying each plot (Panel A through F) in Figure 1.
