# Supplementary material for: AI-Based Triage Decision Support: Multisite Economic Evaluation in the United States
Source: J Med Internet Res. 2026 Jun 3;28:e95213. doi: 10.2196/95213 (PMC13233007; doi:10.2196/95213)
Supplement: Checklist 1 [file jmir-v28-e95213-s002.pdf]

Consolidated Health Economic Evaluation Reporting Standards (CHEERS) Checklist

Topic	No.	Item	Location Reported Page, Section
**Title**			
	1	Identify the study as an economic evaluation and specify the interventions being compared.	Page 1, Title
**Abstract**			
	2	Provide a structured summary that highlights context, key methods, results, and alternative analyses.	Page 1, Abstract
**Introduction**			
**Background and objectives**	3	Give the context for the study, the study question, and its practical relevance for decision making in policy or practice.	Page 2, Introduction
**Methods**			
**Health economic analysis plan**	4	Indicate whether a health economic analysis plan was developed and where available.	Page 3, Economic Model
**Study population**	5	Describe characteristics of the study population (such as age range, demographics, socioeconomic, or clinical characteristics).	Page 2, Study Design and Participants
**Setting and location**	6	Provide relevant contextual information that may influence findings.	Page 2, Study Design and Participants
**Comparators**	7	Describe the interventions or strategies being compared and why chosen.	Page 3, AI Triage CDS Intervention
**Perspective**	8	State the perspective(s) adopted by the study and why chosen.	Page 3, Economic Model
**Time horizon**	9	State the time horizon for the study and why appropriate.	Page 2, Study Design and Participants
**Discount rate**	10	Report the discount rate(s) and reason chosen.	Page 3, Economic Model
**Selection of outcomes**	11	Describe what outcomes were used as the measure(s) of benefit(s) and harm(s).	Page 3, ED Efficiency Outcomes, Economic Model
**Measurement of outcomes**	12	Describe how outcomes used to capture benefit(s) and harm(s) were measured.	Page 4, Revenue Data, Cost and Operating Margin Estimates
**Valuation of outcomes**	13	Describe the population and methods used to measure and value outcomes.	Page 3, Economic Model
**Measurement and valuation of resources and costs**	14	Describe how costs were valued.	Page 3, Economic Model
**Currency, price date, and conversion**	15	Report the dates of the estimated resource quantities and unit costs, plus the currency and year of conversion.	Page 3, Economic Model, Supplemental Table 2
**Rationale and description of model**	16	If modelling is used, describe in detail and why used. Report if the model is publicly available and where it can be accessed.	Page 3, Economic Model
**Analytics and assumptions**	17	Describe any methods for analyzing or statistically transforming data, any extrapolation methods, and approaches for validating any model used.	Page 4, Revenue Data, Cost and Operating Margin Estimates
**Characterizing heterogeneity**	18	Describe any methods used for estimating how the results of the study vary for subgroups.	Page 5, Sensitivity Analyses, Supplemental Table 3
**Characterizing distributional effects**	19	Describe how impacts are distributed across different individuals or adjustments made to reflect priority populations.	Page 3, Economic Model, Supplemental Table 1
**Characterizing uncertainty**	20	Describe methods to characterize any sources of uncertainty in the analysis.	Page 3, Economic Model<br>Page 5, Sensitivity Analyses
**Approach to engagement with patients and others affected by the study**	21	Describe any approaches to engage patients or service recipients, the general public, communities, or stakeholders (such as clinicians or payers) in the design of the study.	Page 7, Implications for Practice
**Results**			
**Study parameters**	22	Report all analytic inputs (such as values, ranges, references) including uncertainty or distributional assumptions.	Page 5, Financial Impact of the AI Triage CDS
**Summary of main results**	23	Report the mean values for the main categories of costs and outcomes of interest and summarise them in the most appropriate overall measure.	Page 5, Financial Impact of the AI Triage CDS
**Effect of uncertainty**	24	Describe how uncertainty about analytic judgments, inputs, or projections affect findings. Report the effect of choice of discount rate and time horizon, if applicable.	Page 6, Economic Model Sensitivity Analyses
**Effect of engagement with patients and others affected by the study**	25	Report on any difference patient/service recipient, general public, community, or stakeholder involvement made to the approach or findings of the study	Page 7, Implications for Practice
**Discussion**			
**Study findings, limitations, generalizability, and current knowledge**	26	Report key findings, limitations, ethical or equity considerations not captured, and how these could affect patients, policy, or practice.	Page 6, Principal Findings, Page 8, Limitations
**Other relevant information**			
**Source of funding**	27	Describe how the study was funded and any role of the funder in the identification, design, conduct, and reporting of the analysis	Page 9, Funding Statement
**Conflicts of interest**	28	Report authors conflicts of interest according to journal or International Committee of Medical Journal Editors requirements.	Page 9, Conflicts of Interest
